# Sensory-specific predictive models in the human anterior insula

**DOI:** 10.12688/f1000research.17961.1

**Published:** 2019-02-06

**Authors:** Gil Sharvit, Patrik Vuilleumier, Corrado Corradi-Dell'Acqua

**Affiliations:** 1 Haas School of Business, University of California, Berkeley, Berkeley, USA; 2 Geneva Neuroscience Center, University of Geneva, Geneva, Switzerland; 3 Laboratory for Behavioural Neurology and Imaging of Cognition, Department of Neuroscience, University of Geneva, Geneva, Switzerland; 4 Theory of Pain Laboratory, Faculty of Psychology and Educational Sciences, University of Geneva, Geneva, Switzerland

**Keywords:** Pain, Expectancy, Nocebo, Bayesian Coding, Unpleasantness

## Abstract

Expectations affect the subjective experience of pain by increasing sensitivity to noxious events, an effect underlain by brain regions such as the insula. However, it has been debated whether these neural processes operate on pain-specific information or on more general signals encoding expectation of unpleasant events. To dissociate these possibilities, two independent studies ( Sharvit *et al.*, 2018, *Pain*; Fazeli and Büchel, 2018, *J. Neurosci*) implemented a cross-modal expectancy paradigm, testing whether responses to pain could also be modulated by the expectation of similarly unpleasant, but painless, events. Despite their differences, the two studies report remarkably convergent (and in some cases complementary) findings. First, the middle-anterior insula response to noxious stimuli is modulated only by expectancy of pain but not of painless adverse events, suggesting coding of pain-specific information. Second, sub-portions of the middle-anterior insula mediate different aspects of pain predictive coding, related to expectancy and prediction error. Third, complementary expectancy effects are also observed for other negative experiences (i.e., disgust), suggesting that the insular cortex holds prospective models of a wide range of events concerning their sensory-specific features. Taken together, these studies have strong theoretical implications on the functional properties of the insular cortex.

One of the most striking breakthroughs in pain research has been the discovery of expectancy modulations, according to which subjective experiences do not only reflect nociceptive input but also individuals’ previous knowledge and beliefs ^1^. Expectancy modulations are noteworthy for their clinical implications, as convincing individuals of the effectiveness of an analgesic might induce a strong pain relief (placebo effect), sometimes comparable to the effects of active agents ^2^. Furthermore, expectancy effects have sparkled a major theoretical debate, with influential models suggesting that pain symptoms might be better explained through a Bayesian framework, where the brain estimates the (posterior) probability of body damage, based on the integration of sensory inputs and prior representations ^3– 6^.

Many studies investigated the neural structures underlying expectancy modulations of pain, pointing to an extensive network including, among other regions, the insular cortex ^1, 7– 10^. In particular, whereas the posterior portion of the insula is known to receive thalamic nociceptive projections ^11– 13^ and thought to process bottom-up components of the painful experience ^8^, the middle-anterior portions may integrate such bottom-up signals with prior expectations ^7, 8^, and generate prediction-error signals, serving to update the representation of future events ^8^. However, the insular cortex (like other interconnected regions such as the cingulate cortex) does not respond to pain specifically, but also to a wide range of aversive events ^14^, including disgust ^15, 16^, negatively-valenced pictures ^17, 18^, or even unfairness ^15, 19, 20^. Accordingly, a part of pain-evoked activity in this region might reflect supramodal dimensions of affect or motivation, such as unpleasantness ^15^, arousal or even salience ^21, 22^. This raises the question about the nature of the predictive information encoded on the middle-anterior insula, and whether it relates to pain-specifically ( *“this will hurt”*), or rather to an undistinctive negative event ( *“this will be bad”*).

Addressing this issue is not a trivial matter, as it would require testing whether pain-evoked activity in the middle-anterior insula is also sensitive to the expectation of a painless event of same unpleasantness or salience. Interestingly, two recent independent studies (each unbeknownst to the other) did precisely this, reaching remarkably similar results ^23, 24^. The first study from Sharvit and others ^23^ compared the expectancy of pain with that of a disgusting odorant of similar unpleasantness (see also Sharvit and others ^25^ for an earlier behavioral implementation of the task), whereas the second from Fazeli and Büchel ^24^ used as control pictures of aversive content. By expanding on well-known paradigms of pain expectancy ^7, 8^, both studies were able to replicate evidence that the middle-anterior insula integrated bottom-up nociceptive information with signals from predictive cues, but this did not occur when cues were incongruent with the subsequent event (e.g., disgust/image cues followed by painful stimulus) ^23, 24^. Such convergence between researches with important differences in sensory stimuli, task structures, and data analyses ^23, 24^, provides a compelling case that expectancy modulations of pain in the insular cortex are sensory-specific, and do not generalize to a broad code of unpleasantness. This also accords with other work showing for shared and segregated portions in insula for representations of pain, disgust, and unfairness ^15^.

Although sharing a similar take-home message, the two cross-modal experiments by Sharvit and Fazeli differ (and in some case complement each other) concerning the information coded by the middle-anterior insula. By employing rigorous Bayesian modelling, Fazeli and Büchel ^24^ dissociated a portion in the middle and dorsal-anterior portion of the insula, responsible for integrating bottom-up signals with prior expectancies, from a portion in ventral-anterior insula, responsible for generating error signals whenever the painful stimulus greatly diverged from what was predicted by the cues (see Figure 1). This was not the case in Sharvit and others ^23^ who adopted a paradigm where divergences between cues and subsequent stimuli were purposefully subtle to pass unnoticed ^7, 25^. It is interestingly to notice, however, that Sharvit and others ^23^ reported a dissociation between the middle insula, exerting a mediatory role in the way in which predictive cues influenced subjective reports (as previously found ^7^), and the most anterior insula, exerting instead an opposite role of suppression. Hence, in Sharvit and others ^23^ activity in the anterior insula seemed to prevent individuals from being influenced by their expectations, an effect that is consistent with the notion of prediction-error modeled by Fazeli and Büchel ^24^. The two studies also differ regarding the insular sub-sections involved: Sharvit and others ^23^ mapped mediation and suppression effects along the middle-to-anterior axis, whereas Fazeli and Büchel ^24^ described expectancy and prediction-error effects also along the dorsal-to-ventral axis of the anterior insula ( Figure 1). Future studies will need to further clarify how different components of expectancy relate to the various insula portions.

**Figure 1.**
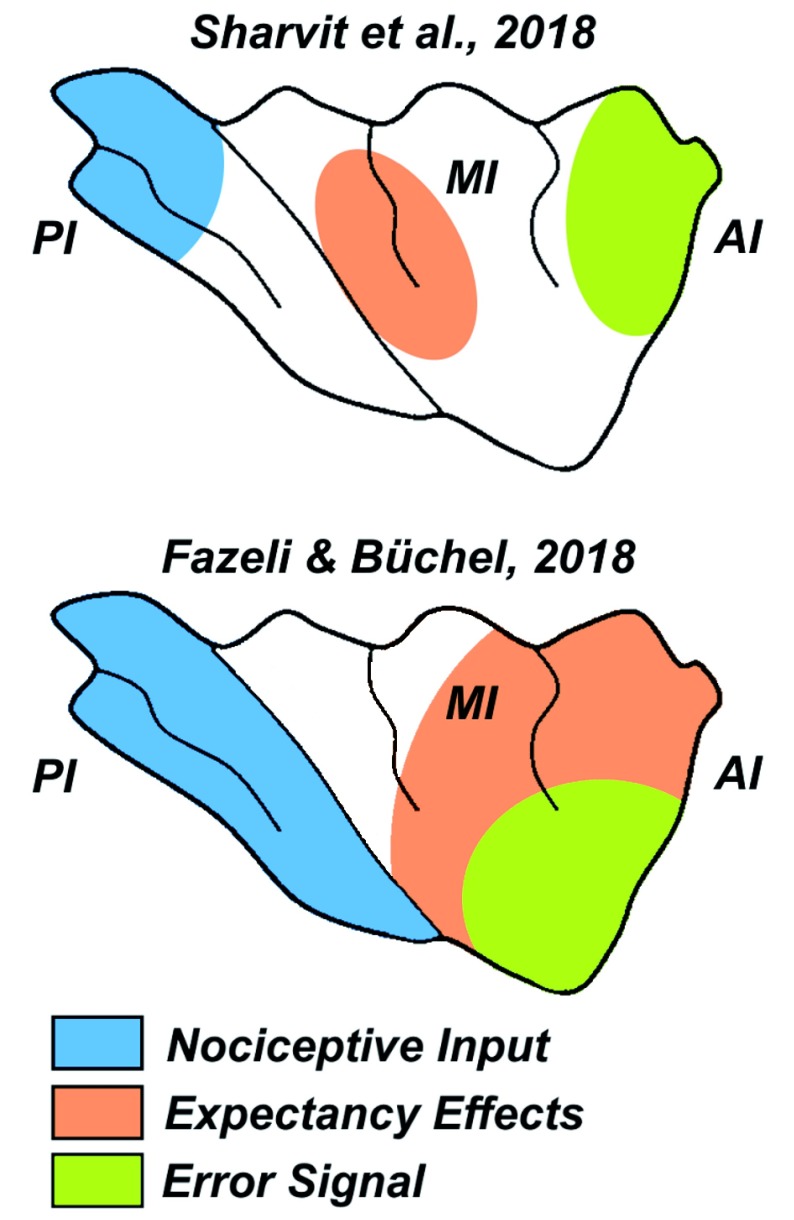
Schematic representation of sensory-specific expectancy processing in the insular cortex from the studies of Sharvit and others ^23^ and Fazeli and Büchel ^24^. Blue shades over the Posterior Insula (PI) refer to processing of pain based solely on nociceptive inputs. Orange shades over the Middle (MI) and Anterior Insula (AI), refer to processing of pain (and disgust ^23^) based also on prior expectations. Green shades in AI refer to regions coding prediction errors ^24^, and suppressing the effect of previous expectations ^23^.

A further, and critical, point of divergence relates to whether the insular cortex is also susceptible to sensory-specific expectancy for other events than pain. This question was addressed only by Sharvit and others ^23^ who described complementary effects to those observed in pain, also for the case of olfactory disgust. These results suggested that the middle-anterior insula may hold multiple predictive representations of upcoming events, which are then updated by bottom-up sensory input. Hence, although the middle-anterior insula appears sensitive to a wide range of stimuli ^14^, it may retain sensory-specific information about each of them. Anatomical studies on primates subfields in this region ^26^, with a level of detail that exceeds that derived from neuroimaging research in humans ^27, 28^. It is therefore foreseeable that different kinds of sensory events might be represented in the anterior insula through neighbouring, but distinct, neuronal populations, which could be difficult to distinguish through radiological imaging, but nonetheless selectively dissociated through well-crafted expectancy manipulations.

## Data availability

All data underlying the results are available as part of the article and no additional source data are required.
