# Supplementary material for: Sensory-specific predictive models in the human anterior insula
Source: F1000Res. 2019 Feb 6;8:164. [Version 1] doi: 10.12688/f1000research.17961.1 (PMC6402078; doi:10.12688/f1000research.17961.1)
Supplement: Peer review history

# Peer review: Reviewer response for version 1

**DOI:** 10.5256/f1000research.19643.r44441

**Published:** 2019-03-01

**Authors:** Daniele Romano

**Affiliations:** 1 Department of Psychology, University of Milano Bicocca, Milan, Italy

The paper by Sharvit and colleagues is an interesting commentary paper mostly based on two studies aiming at exploring the role of the insular cortex in integrating sensory input and internal predicting models to formulate the perception of an aversive stimulus (Fazeli and Büchel (2018 ^1^) and Sharvit *et al.* (2018 ^2^)). Sharvit and co-workers also wrote one of the two papers on which this commentary is based.

 The two original studies focused on comparing the response to painful stimuli to another negative stimulation. In one case a disgusting olfactory stimulation was used, while in the other, a set of negative-valence pictures from the IAPS database were used.

 The commentary paper nicely points out the convergences of the two studies and their potential limitations. It also importantly noticed the differences in the two findings discussing their convergences and the complementary nature of the two. I have no significant concerns about the commentary paper, and I believe that this offers an interesting supplement to the original discussion of Sharvit and colleagues’ work.

 I only wish to point out a few comments to further develop and hopefully stimulate the discussion about the potential role of the insula in predictive coding.

1. It is known that it can be difficult to match the saliency of negative stimuli with the saliency of positive stimuli. The former are typically more arousing than the latter (Ferri *et al.* (2015 ^3^)). However, when this aspect is taken into consideration carefully, results (at least at the behavioural level) show that saliency matters more than valence (Spaccasassi *et al.* (2019 ^4^)). Although it is clearly the aim of the two original studies, is it possible that the insula contributes to the predictive coding of every salient stimulus? In other words, is it possible that the role of the insula is not limited to the two hypotheses “this will hurt” or “this will be bad”, but may extend to “this will be arousing”?
2. Relatedly, an even more extreme hypothesis could be made. The insula might be a sort of bottle-neck for the integration of internal models with sensory stimuli. Returning to the previous example, it is possible that the insula processing is the following: “are the sensory information and the internal models on the same page? Can I integrate the two sources of information?” The middle and anterior insula might be involved in the processing of this very general aspect. Sharvit and Fazeli papers mostly work in the prediction of stimuli of different sensory modalities with a similar intensity and the same negative valence, revealing a crucial role of the middle and anterior insula in coding prediction errors across sensory modalities. Potentially, it is possible to create a prediction incompatible with the incoming stimulus, so that it won’t be integrated, within a sensory modality. For example, the prediction of a very low painful stimulus coupled with a very high level of painful stimuli may generate the same prediction error signal that leads to the non-integration of the internal/external signals when coming from different sensory modalities. Fazeli and Büchel (2018 ^1^) already proposed this point as a limitation of their study in their discussion.
3. There is a debate that social exclusion may generate what is called “social pain”, which would have a brain footprint largely overlapping with physical pain (Eisenberger and Lieberman (2004 ^5^)). I am not personally a great sponsor of this “overlap theory”, the difference between the two experiences is immense perceptually so that the overlap between the two functions should be taken cautiously. Besides my personal concerns, one hypothesis is that with current experimental procedures we capture effects that are shared by all the arousing stimuli and not limited to pain (Legrain *et al.* (2011 ^6^)). Do the authors predict that the role of the insula in integrating expectations and sensory input would somehow work also for social pain? Alternatively, can the sensory-specific pattern of response for expectations, discussed in this commentary, help to distinguish the processing of physical pain from social pain?

I have read this submission. I believe that I have an appropriate level of expertise to confirm that it is of an acceptable scientific standard.

## References

[1] FazeliSBüchelC: Pain-Related Expectation and Prediction Error Signals in the Anterior Insula Are Not Related to Aversiveness. *J Neurosci*.2018;38(29) : 10.1523/JNEUROSCI.0671-18.2018 6461-6474 10.1523/JNEUROSCI.0671-18.2018 29934355PMC6705956

[2] SharvitGCorradi-DellʼAcquaCVuilleumierP: Modality-specific effects of aversive expectancy in the anterior insula and medial prefrontal cortex. *Pain*.2018;159(8) : 10.1097/j.pain.0000000000001237 1529-1542 10.1097/j.pain.0000000000001237 29613910

[3] FerriFTajadura-JiménezAVäljamäeAVastanoRCostantiniM: Emotion-inducing approaching sounds shape the boundaries of multisensory peripersonal space. *Neuropsychologia*.2015;70: 10.1016/j.neuropsychologia.2015.03.001 468-75 10.1016/j.neuropsychologia.2015.03.001 25744869

[4] SpaccasassiCRomanoDMaravitaA: Everything is worth when it is close to my body: How spatial proximity and stimulus valence affect visuo-tactile integration. *Acta Psychol (Amst)*.2019;192: 10.1016/j.actpsy.2018.10.013 42-51 10.1016/j.actpsy.2018.10.013 30412839

[5] EisenbergerNILiebermanMD: Why rejection hurts: a common neural alarm system for physical and social pain. *Trends Cogn Sci*.2004;8(7) : 10.1016/j.tics.2004.05.010 294-300 10.1016/j.tics.2004.05.010 15242688

[6] LegrainVIannettiGDPlaghkiLMourauxA: The pain matrix reloaded: a salience detection system for the body. *Prog Neurobiol*.2011;93(1) : 10.1016/j.pneurobio.2010.10.005 111-24 10.1016/j.pneurobio.2010.10.005 21040755

# Peer review: Reviewer response for version 1

**DOI:** 10.5256/f1000research.19643.r44134

**Published:** 2019-03-01

**Authors:** Lauren Y. Atlas, In-Seon Lee

**Affiliations:** 1 National Institute on Drug Abuse, National Institutes of Health, Baltimore, MD, USA; 2 National Center for Complementary and Integrative Health, National Institutes of Health, Bethesda, MD, USA

In this paper, Sharvit, Vuilleumier and Corradi-Dell’Acqua draw on evidence from two recent studies (Sharvit *et al.* (2018 ^1^) and Fazeli and Büchel (2018 ^2^)) to suggest that the middle-anterior part of the insula cortex encodes sensory-specific expectancy effects of pain while the posterior insula process nociceptive inputs. Both investigations employ within-subjects designs to examine whether the brain shows sensory-specific expectancy effects. Cues predict the intensity or unpleasantness of pain or a different modality (olfactory or aversive images), and cues about one modality are occasionally followed by the other outcome. The authors of each paper make conclusions about cross-modal versus sensory-specific effects, which are summarized in the current review with a focus on effects within the insula. The paper is well-written and covers important issues regarding whether the insula processes expectation of general unpleasantness or pain-specific expectation. This question is timely and relevant for a large body of work asking whether brain responses are specific to pain or whether responses in regions like the insula reflect domain-general processes, such as salience or unpleasantness (Legrain *et al.* (2011 ^3^). The review is a useful addition to the literature on pain and sensory specificity. However, certain issues that should be addressed in this review to benefit future investigations on the question of expectancy effects and modality specificity.

 In our opinion, the authors need to be more cautious in making conclusions regarding sensory specificity since neither study included comprehensive tests for specificity. First, they did not evaluate potentially confounding factors between pain and non-pain stimuli such as stimulus-related salience, arousal, uncertainty (the degree of learned association between cue and outcome), and threat/aversiveness. For example, a modality-specific expectancy effect in the anterior insula might be influenced by the lower level of uncertainty in the congruent conditions compared to incongruent conditions. The insula is also a core region of the salience network and affected by the salience of the stimulus (Menon and Uddin (2010 ^4^)) as well as threat value (Wiech *et al.* (2010 ^5^)) which might differ between pain and the other modality (i.e. pain has the potential to cause harm, whereas odors do not). Since these factors were not measured or controlled in either study, it should be noted that those factors might contribute to the effect of expectancy in the insula as well.

 Second, Fazeli and Büchel (2018 ^2^) did not evaluate sensory-specific or sensory-general processing of expectancy in response to visual stimuli paired with either pain or visual cues. If the effect of expectancy on insula activity is truly sensory-specific and does not affect the expectancy effect on a stimulus of another modality, the same phenomenon should be found in the non-pain modality. Finally, Sharvit *et al.* (2018 ^1^) trained with outcome-specific cues during conditioning, and tested responses to crossed cues only in the test phase. This may have led to differences in the cross-modal cue pairs, only because those were novel in the test phase, which is also related to the issue of uncertainty mentioned above. 

 Finally, we think the authors should also consider that within-subjects tests of sensory specificity may lead to very different conclusions from between-subjects designs, wherein subjects would be randomly assigned to experience a single outcome modality and modalities would vary across groups. When individuals experience multiple outcomes, this engenders both value-based learning (expectations about intensity/unpleasantness) and sensory learning (expectations about outcome identity). Some regions respond similarly to both types of learning (e.g. dopaminergic neurons involved in value-based prediction error respond when outcome identity changes and value is held constant (Takahashi *et al.* (2017 ^6^) and Chang *et al.* (2017 ^7^)), whereas other brain regions respond to value irrespective of outcome identity (OFC; Padoa-Schioppa *et al.* (2008 ^8^)) or sensory outcome irrespective of value (lateral OFC; Boorman *et al.* (2016 ^9^)). In the two papers reviewed here, cues denote both outcome type (i.e. pain vs. odor/image) and outcome value (i.e. unpleasantness/intensity), which requires both types of learning. While this is theoretically interesting, the question Sharvit and co-authors focus on here is whether **value-based learning (i.e. expectancy-based modulation) is sensory specific**. A purer test of this question would be to use a between-subjects design to compare value-based processing across modalities, i.e. whether brain responses are similar when pain is preceded by pain expectancy cues and when olfactory/visual outcomes are paired with expectancy cues in those domains. When both forms of learning are combined, differences might emerge that reflect sensory learning, rather than purely testing whether value-learning is sensory-specific. The authors should acknowledge this alternative in the present review.

 In addition to the two conceptual issues above, we have several minor suggestions:

1. Several terms should be defined or explained more precisely (either in a box or early in the manuscript). This includes: “sensory specificity”; the distinction between ‘unpleasant events’, ‘negative experiences’, and ‘threat’; and the relationship between Sharvit’s manipulation of  ‘unpleasantness’ (Sharvit *et al.* (2018 ^1^)) and Fazeli’s manipulation of ‘intensity’ (Fazeli and Büchel (2018 ^2^)). 
2. The manuscript would benefit from more description of the two original study paradigms and how they differ (e.g. conditioning, instructions, test phases). 
3. The review focuses on findings in the insula, yet both papers also found important effects outside of this region (Sharvit *et al.* (2018 ^1^) found interesting and similar cue predictive effects in vmPFC and TPJ, and Fazeli and B üchel (Fazeli and Büchel (2018 ^2^)) showed intensity and expectancy effects in ACC in addition to the insula). Since insula has a strong relationship (functional and anatomical) with those regions in pain processing and modulation, the authors should acknowledge that other regions show similar effects and also discuss differences.

We have read this submission. We believe that we have an appropriate level of expertise to confirm that it is of an acceptable scientific standard.

## References

[1] SharvitGCorradi-DellʼAcquaCVuilleumierP: Modality-specific effects of aversive expectancy in the anterior insula and medial prefrontal cortex. *Pain*.2018;159(8) : 10.1097/j.pain.0000000000001237 1529-1542 10.1097/j.pain.0000000000001237 29613910

[2] FazeliSBüchelC: Pain-Related Expectation and Prediction Error Signals in the Anterior Insula Are Not Related to Aversiveness. *J Neurosci*.2018;38(29) : 10.1523/JNEUROSCI.0671-18.2018 6461-6474 10.1523/JNEUROSCI.0671-18.2018 29934355PMC6705956

[3] LegrainVIannettiGDPlaghkiLMourauxA: The pain matrix reloaded: a salience detection system for the body. *Prog Neurobiol*.2011;93(1) : 10.1016/j.pneurobio.2010.10.005 111-24 10.1016/j.pneurobio.2010.10.005 21040755

[4] MenonVUddinLQ: Saliency, switching, attention and control: a network model of insula function. *Brain Struct Funct*.2010;214(5-6) : 10.1007/s00429-010-0262-0 655-67 10.1007/s00429-010-0262-0 20512370PMC2899886

[5] WiechKLinCSBrodersenKHBingelUPlonerMTraceyI: Anterior insula integrates information about salience into perceptual decisions about pain. *J Neurosci*.2010;30(48) : 10.1523/JNEUROSCI.2087-10.2010 16324-31 10.1523/JNEUROSCI.2087-10.2010 21123578PMC6634837

[6] TakahashiYKBatchelorHMLiuBKhannaAMoralesMSchoenbaumG: Dopamine Neurons Respond to Errors in the Prediction of Sensory Features of Expected Rewards. *Neuron*.2017;95(6) : 10.1016/j.neuron.2017.08.025 1395-1405.e3 10.1016/j.neuron.2017.08.025 28910622PMC5658021

[7] ChangCYGardnerMDi TillioMGSchoenbaumG: Optogenetic Blockade of Dopamine Transients Prevents Learning Induced by Changes in Reward Features. *Curr Biol*.2017;27(22) : 10.1016/j.cub.2017.09.049 3480-3486.e3 10.1016/j.cub.2017.09.049 29103933PMC5698141

[8] Padoa-SchioppaCAssadJA: The representation of economic value in the orbitofrontal cortex is invariant for changes of menu. *Nat Neurosci*.2008;11(1) : 10.1038/nn2020 95-102 10.1038/nn2020 18066060PMC2646102

[9] BoormanEDRajendranVGO'ReillyJXBehrensTE: Two Anatomically and Computationally Distinct Learning Signals Predict Changes to Stimulus-Outcome Associations in Hippocampus. *Neuron*.2016;89(6) : 10.1016/j.neuron.2016.02.014 1343-1354 10.1016/j.neuron.2016.02.014 26948895PMC4819449
